# Minimally invasive intrathoracic anastomosis after Ivor Lewis esophagectomy for cancer: a review of transoral or transthoracic use of staplers

**DOI:** 10.1007/s00464-012-2149-z

**Published:** 2012-02-01

**Authors:** K. W. Maas, S. S. A. Y. Biere, J. J. G. Scheepers, S. S. Gisbertz, V. Turrado Rodriguez, D. L. van der Peet, M. A. Cuesta

**Affiliations:** Department of Surgery, VU University Medical Center, De Boelelaan 1117, 1081 HV Amsterdam, The Netherlands

**Keywords:** Oesophageal, Thoracoscopy, Esophagus, Ivor Lewis, Intrathoracic, Anastomosis, Minimally invasive

## Abstract

**Background:**

Minimally invasive Ivor Lewis esophagectomy is one of the approaches used worldwide for treating esophageal cancer. Optimization of this approach and especially identifying the ideal intrathoracic anastomosis technique is needed. To date, different types of anastomosis have been described. A literature search on the current techniques and approaches for intrathoracic anastomosis was held. The studies were evaluated on leakage and stenosis rate of the anastomosis.

**Methods:**

The PubMed electronic database was used for comprehensive literature search by two independent reviewers.

**Results:**

Twelve studies were included in this review. The most frequent applied technique was the stapled anastomosis. Stapled anastomoses can be divided into a transthoracic or a transoral introduction. This stapled approach can be performed with a circular or linear stapler. The reported anastomotic leakage rate ranges from 0 to 10%. The reported anastomotic stenosis rate ranges from 0 to 27.5%.

**Conclusions:**

This review has found no important differences between the two most frequently used stapled anastomoses: the transoral introduction of the anvil and the transthoracic. Clinical trials are needed to compare different methods to improve the quality of the intrathoracic anastomosis after esophagectomy.

Despite the important development of the minimally invasive approach of esophageal cancer, esophagectomy is still associated with a significant risk of perioperative morbidity [1]. After a successfully performed esophageal resection, the creation of a safe anastomosis is essential to reduce the risk of leakage and related complications.

There is an important trend to anastomose the gastric tube with the intrathoracic esophagus, the so-called Ivor Lewis operation [2]. Risk for anastomotic leakage in the thorax with possibly fatal consequences has resulted in the development of the three-stage approach with a cervical anastomosis by McKeown [3], and the transhiatal approach with a cervical anastomosis by Orringer and Sloan [4]. In case of anastomotic leakage in the neck, a subsequent cervical fistula is a manageable complication [5]. There is, however, some evidence that cervical anastomosis could be related to more anastomotic leakage and stenosis [6]. Currently, the increased numbers of gastroesophageal junction tumors form an ideal indication for an Ivor Lewis procedure. This approach may reduce recurrent nerve lesion and other complications associated with a cervical dissection. Moreover, a shorter gastric conduit will permit a more extended gastric resection and will, because of a good vascular supply, lead to less anastomotic leakages. This transthoracic procedure may be performed by a conventional or minimally invasive approach.

The question is which type of intrathoracic anastomosis is the ideal. According to the review of Blackmon et al. [7], stapled anastomosis in conventional surgery will have less leakages and stenosis than the manual anastomosis.

In this paper, all different techniques used for intrathoracic anastomosis in minimally invasive Ivor Lewis esophagectomy have been reviewed showing small differences concerning leakages and stenosis. Due to the increased implementation of the use of minimally invasive surgery for esophageal resection and the number of distal adenocarcinomas and gastroesophageal junction tumors, the ideal anastomosis has still to be found.

## Methods

The PubMed electronic database was used for literature search. A comprehensive search was performed using the following terms: esophagus, esophagectomy, anastomosis, intrathoracic, cancer, Ivor Lewis, minimally invasive, MIE, laparoscopy, thoracoscopy, and esophagogastric. Related terms and combinations also were used (e.g., thoracic, esophagectomy).

Relevant titles were identified and abstracts were read to decide eligibility. When the information in the title and abstract met the objectives of this review, the full article was read. A manual cross-reference search of the references of the relevant articles was performed to identify studies beside the computerized search. Furthermore, the “related articles” feature of PubMed was used. Two reviewers (KWM and SSAYB) executed the search independently of each other.

## Results

**Fig. 1:**
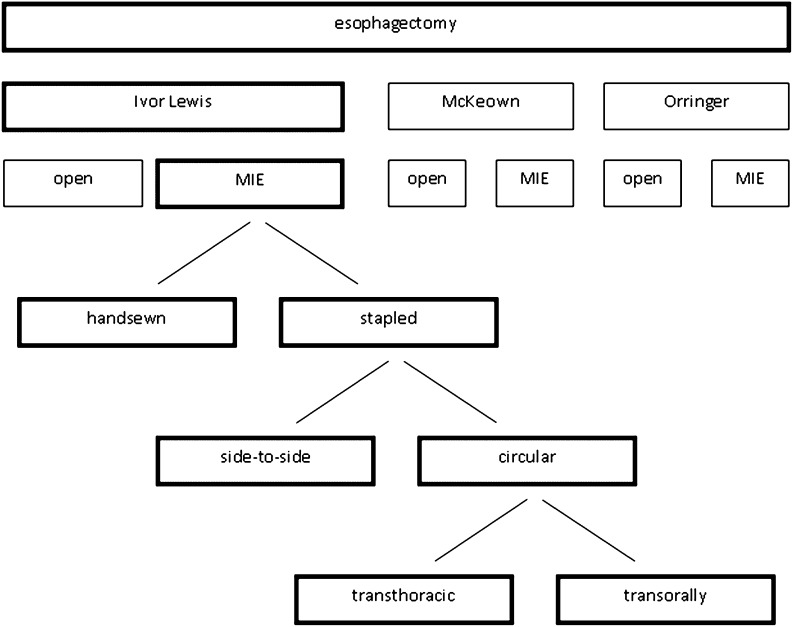
Overview of minimally invasive anastomosis techniques

Intrathoracic anastomoses can be accomplished by a handsewn or stapler technique (Fig. 1). Twelve studies were included in this review to analyze the different techniques. Tables 1 and 2 depict the study characteristics, anastomotic outcomes, and complications. The reported anastomotic leakage rate ranges from 0 to 10% and the anastomotic stenosis from 0 to 27.5%, showing no differences between the type of anastomosis. Compared with the open Ivor Lewis operation, the range of complications were 0–4% for anastomotic leakage and 14.3–28.6% for anastomotic stenosis [8, 9].

**Table 1:** Study characteristics

Study	No. patients	Surgical approach	Patients position^a^	Anastomotic technique	Type of anastomosis	Layers	Fixation of anvil
Watson et al. (1999) [10]	2	Transthoracic	Prone position	Handsewn	End-to-side	Single layer	NA
Cadiere et al. (2010) [11]	1	Transthoracic	Prone position	Handsewn	Side-to-end	Two layers	NA
Lee et al. (1997) [12]	8	Transhiatal and transthoracic	45° Semilateral position	Circular stapled	End-to-side	NA	Pursestring
Nguyen et al. (2001) [15]	1	Transthoracic	Left lateral decubitus position	Circular stapled	End-to-side	NA	Handsewn pursestring
Misawa et al. (2005) [17]	5	Transthoracic	Left lateral decubitus position	Circular stapled	End-to-side	NA	Pursestring Endo-stitch
Bizekis et al. (2006) [18]	50	Transthoracic	Left lateral decubitus position	Circular stapled	End-to-side	NA	NR
Thairu et al. (2007) [19]	18	Transthoracic	Prone position	Circular stapled	End-to-side	NA	Linear staple gun and Z-stitch
Sutton et al. (2002) [21]	10	Transhiatal	Supine position	Transorally circular stapled	End-to-side	NA	NR
Nguyen et al. (2008) [22]	51	Transthoracic	Left lateral decubitus position	Transorally circular stapled	End-to-side	NA	NR
Campos et al. (2010) [23]	37	Transthoracic	NR	Transorally circular stapled	End-to-side	NA	NR
Ben-David et al. (2010) [24]	6	Transthoracic	Left lateral decubitus position	Linear stapled	Side-to-side	NA	NR
Gorenstein et al. (2011) [25]	31	Transthoracic	Left lateral decubitus position	Linear stapled	Side-to-side	NA	NR

^a^During performance of anastomosis

*NA* not applicable; *NR* not reported

**Table 2:** Anastomotic outcome in minimally invasive and open Ivor Lewis esophagectomy

Study	No. patients	Anastomotic leak	Anastomotic stenosis
Watson et al. (1999) [10]	2	0	0
Cadiere et al. (2010) [11]	1	0	0
Lee et al. (1997) [12]	8	0	1 (12.5%)
Nguyen et al. (2001) [15]	1	0	0
Misawa et al. (2005) [17]	5	0	0
Bizekis et al. (2006) [18]	50	3 (6%)	6 (12%)
Thairu et al. (2007) [19]	18	0	Not reported
Sutton et al. (2002) [21]	10	1 (10%)	Not reported
Nguyen et al. (2008) [22]	51	5 (9.8%)	14 (27.5%)
Campos et al. (2010) [23]	37	1 (2.7%)	5 (13.5%)
Ben-David et al. (2010) [24]	6	Not reported	0
Gorenstein et al. (2011) [25]	31	1 (3.2%)	Not reported
Chasseray et al. (1989)^a^ [8]	49	2 (4%)	7 (14.3%)
Walther et al. (2003^a^) [9]	42	0	12 (28.6%)

^a^Conventional open esophagectomy

### Handsewn technique

The first description of a totally endoscopic Ivor Lewis esophagectomy with an intrathoracic anastomosis was reported in 1999 by Watson et al. [10]. The thoracoscopic phase of the operation was performed in the prone position. They described two patients in which the intrathoracic anastomosis was achieved with a handsewn single-layer technique. Both patients recovered without complications and with a short hospital stay.

**Fig. 2:**
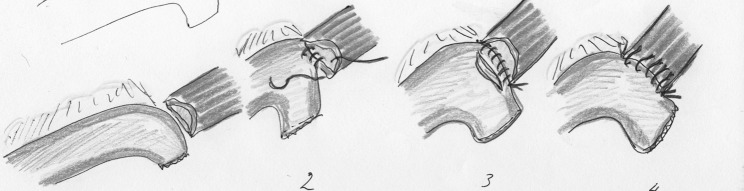
Handsewn intrathoracic anastomosis (in line illustrations by M.A. Cuesta)

Ten years later, Cadiere et al. [11] described a totally minimally invasive Ivor Lewis esophagectomy with handsewn anastomosis. The patient was operated through right thoracoscopy in the prone position and laparoscopy. No postoperative complications were reported in this case report. An illustration of the handsewn intrathoracic anastomosis technique is shown in Fig. 2.

### Stapled technique

#### Transthoracic circular stapled anastomosis

**Fig. 3:**
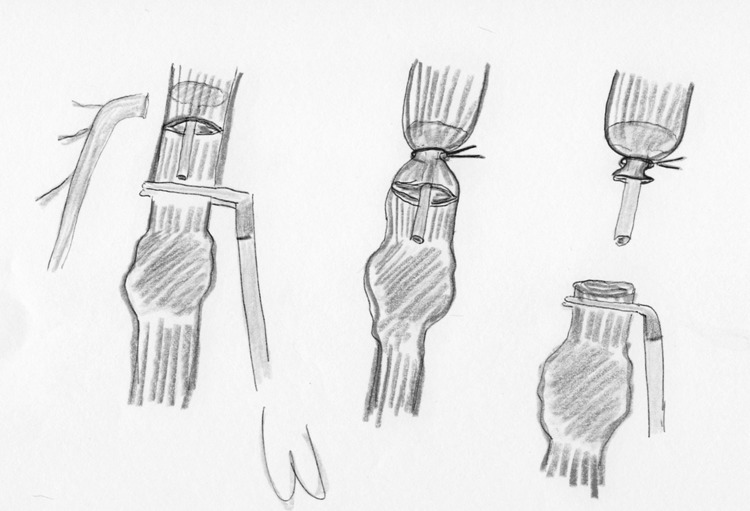
Fixation of the anvil by a Roeder’s Knot (in line illustrations by M.A. Cuesta)

In 1997, Lee et al. [12] described a one-stage right lateral thoracoscopic esophagectomy with intrathoracic stapled anastomosis in a series of eight patients with carcinoma of the lower esophagus. The operation was performed with a double-lumen tracheal tube to enable collapse of the right lung. The patient was placed in 45  position with the right side up. A two-team synchronous approach was used: one for the abdominal stage to perform the mobilization of the stomach through a transverse incision laparotomy, and the other team performed the thoracoscopic approach. Esophagogastric anastomosis was fashioned by a stapling device using the ligature method described by Allsop and Ng [13, 14]. A right-angled clamp inserted by the abdominal surgeon through the hiatus was applied onto the esophagus just proximal to the lesion. A transverse incision in the esophagus was placed, with adequate margin, above the tumor. The abdominal surgeon inserted the anvil (28-mm circular stapler) into the thoracic cavity through the hiatus and introduced it into the lumen of the esophagus. A Vicryl ligature was applied around the esophagus by means of Roeder’s knot to fix the anvil in the proper position and the esophagus divided as illustrated in Fig. 3. The cardioesophageal junction was transacted with a GIA linear stapler and through a transverse gastrostomy the circular stapler was inserted, the stomach introduced into the thorax, and the anastomosis performed. The operation was successful in seven patients, and the remaining patient required conversion to thoracotomy. No leakages were recorded. On short term, one patient developed a benign stenosis that required dilatation.

The anastomosis technique described by Nguyen et al. in 2001 [15] in a case report was performed through a right lateral thoracoscopy with lung block. After the laparoscopic phase, the esophagus was dissected by thoracoscopy and divided with the stapler 2 cm below the azygos vein. The esophageal specimen was retrieved through an enlarged (4 cm) posterior trocar site. Using this small incision, the anvil of 21- or 25-mm circular stapler was placed into the chest and inserted through the opening of the esophagus and secured with a handsewn pursestring suture. The circular stapler was introduced into the chest and passed through an anterior gastrostomy of the gastric conduit. A stapled end-to-side esophagogastric anastomosis was made. Most of the techniques described for circular stapled anastomosis are similar to this technique [16].

**Fig. 4:**
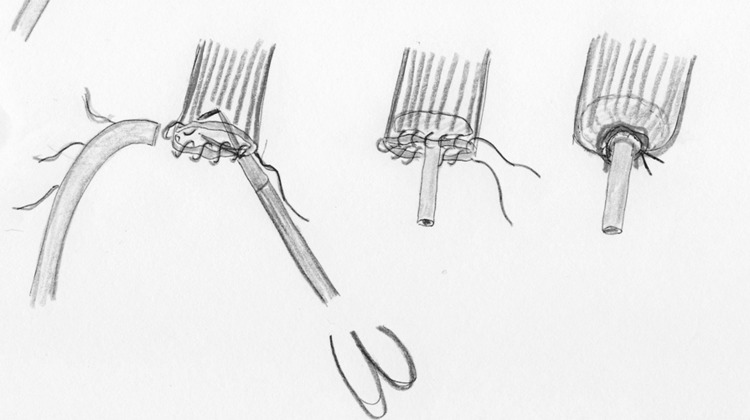
Fixation of the anvil by purse-string using the Endostitch (in line illustrations by M.A. Cuesta)

During the next years, new techniques have been described to facilitate the placement of the anvil in the proximal esophagus. Misawa et al. [17] approached the esophagus, after the laparoscopic phase, through a lateral right thoracoscopy in five patients with cancer of the middle and lower thoracic esophagus. A 5-cm-long thoracotomy was made on the fifth intercostal space. After mobilization of the esophagus, the level of transection was decided and the distal esophagus tied with a 2-0 ligature. The esophageal wall was anteriorly opened and two clamps grasped the esophageal edges allowing the Endo-Stitch^®^ (US Surgical) device for achieving a pursestring suture. After this, the anvil of a 25-mm circular stapler was introduced into the proximal esophagus and the pursestring tied. An Endoloop^®^ ligature was placed over the pursestring to reinforce it (Fig. 4). After retrieval of the distal esophagus with the lesion, the proximal end of the gastric tube was pulled out of the minithoracotomy and the circular stapler was introduced through it into a gastrostomy incision. No leakage or other postoperative complications were observed after this procedure.

Bizekis et al. [18] reported their experience with minimally invasive Ivor Lewis esophagectomy in 50 patients, the majority with tumors with extension into the cardia or a gastroesophageal junction tumor. Thirty-five patients underwent a hybrid Ivor Lewis procedure, defined as laparoscopic gastric mobilization combined with a 5-cm minithoracotomy, and the other 15 underwent the total minimally invasive approach. In the total minimally invasive procedure, after mobilization the esophagus was transacted 2–3 cm above the azygos vein, and an inferior intercostal port site was enlarged to allow the introduction of the circular stapler and for retrieval of the specimen. A 25-mm anvil was placed into the proximal esophagus and secured by means of a pursestring suture (Endo-stitch, US Surgical). The stapler was introduced into the gastric conduit and a circular anastomosis was created in a side-to-end fashion. The redundant gastric conduit was removed by using a linear stapler. In the hybrid procedure, after the laparoscopic phase, the patient was placed in the lateral position and a 5-cm minithoracotomy performed. A rib retractor was placed and the thoracic esophagus mobilized under direct vision. A stapled esophagogastric anastomosis was created in the same fashion. There were three anastomotic leaks in the hybrid group: one empyema not related to leak, one chylothorax, and one pulmonary embolism. They observed 6% mortality. Moreover, six patients developed stenosis of the anastomosis, being dilated postoperatively.

To obviate the necessity of the placement of a pursestring suture or a manually tied knot to secure the anvil, Thairu et al. [19] described a technique in which the head of the anvil was inserted through the anterior wall of the esophagus, opened with dissecting scissors. A linear staple was fired at 60° to the longitudinal axis first right and after left to the spike of the anvil thus forming a V. Around this aperture a Z-stitch was placed, which secured the anvil in place, followed by an end-to-side anastomosis. Using this technique in 18 patients, they observed no anastomotic leaks postoperatively. No midterm results were reported (e.g., stenosis).

#### Transoral circular stapled anastomosis

An important development is the introduction of the anvil transorally into the proximal esophagus, as described originally by Wittgrove et al. [20] for the gastrojejunostomy construction of the gastric bypass in bariatric surgery after the initial work of Sutton et al. in 2002 using a self-adopted circular anvil system [21].

**Fig. 5:**
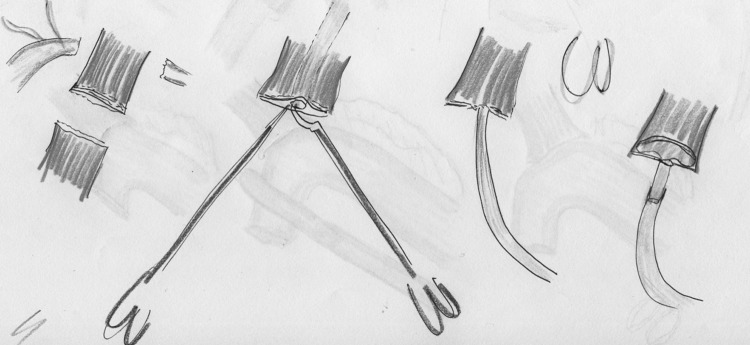
Transoral introduction of anvil by gastric tube (in line illustrations by M.A. Cuesta)

Nguyen et al. [22] described the transoral technique in a series of ten patients to perform the intrathoracic anastomosis after esophageal resection. A commercially available prepared pretilted anvil’s head tip was attached to an oral-gastric tube that was given to the anesthesiologist. The tilted configuration of the anvil improved the ease of transoral passage. After the transection of the esophagus by means of linear stapler, the tube was passed transorally until it was felt within the proximal esophageal stump. A small opening at this level was made perpendicular to the staple line and the tube was advanced through it and withdrawn until the anvil was in the right position at the end of the esophageal stump. The oral-gastric tube was removed after cutting the suture that attached it to the anvil. The head of the anvil returned to the flat position when attached to the spike of the 25-mm circular stapler to perform the anastomosis (Fig. 5). The authors applied the technique without passage problems in ten patients. Moreover, tissue donuts were complete in all cases and there were no postoperative leaks.

Campos et al. [23] confirmed the good results using the transoral anvil technique in 37 patients with a distal esophageal cancer. After the laparoscopic approach in 81% of the patients, the thoracic portion was performed using a muscle-sparing minithoracotomy in 23 patients (62%) and total thoracoscopic approach in 14 patients (37%). There were no intraoperative technical failures of the anastomosis or deaths. Five patients developed strictures (13.5%), and all were successfully treated with endoscopic dilatations. One patient had an anastomotic leak (2.7%) that was treated by reoperation and endoscopic stenting of the anastomosis.

#### Side-to-side stapled anastomosis

Ben-David et al. [24] described in 2010 six patients with gastroesophageal junction cancers in whom after laparoscopic dissection and formation of the gastric conduit, the thorax was approached through a lateral right thoracoscopy. After dissection, the esophagus was divided at the level of the azygos vein using a 60-mm stapler. The transacted proximal esophagus and gastric conduit were aligned with sutures. An esophagostomy was created at the distal end of the transacted esophagus and a gastrostomy performed proximal of the end of the gastric conduit. With the aid of traction sutures, a side-to-side 6-cm linear stapled esophagogastrostomy was performed. After this, the common opening was closed with a running suture. There were no leakages of anastomosis or other postoperative complications. At a median follow-up of 9 months, no postoperative strictures were reported.

**Fig. 6:**
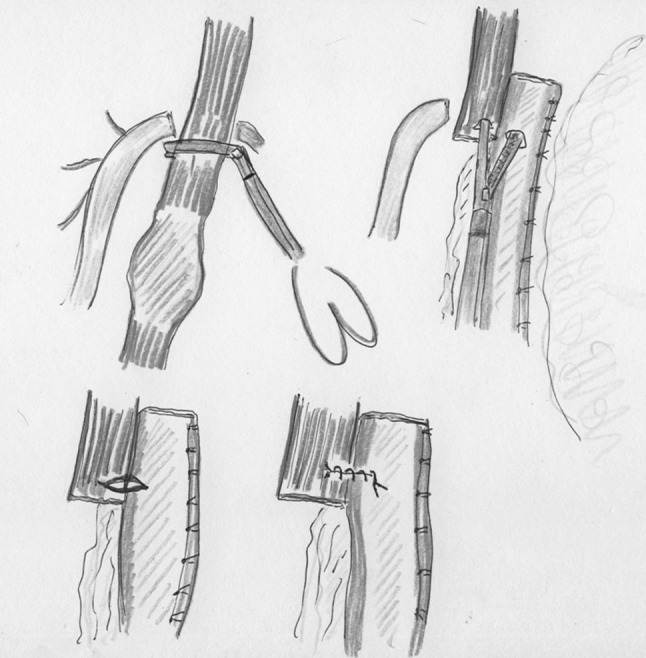
Side-to-side stapled intrathoracic anastomosis (in line illustrations by M.A. Cuesta)

Gorenstein et al. [25] described a slight different side-to-side anastomosis technique in which the proximal esophagus was not stapled, and used the whole lumen for the construction of the side-to-side anastomosis by means of a linear stapler. Once a 4-cm anastomosis was constructed, the anterior defect was closed. Of the 31 patients operated on with this approach, one developed leakage that required reoperation. There were no other anastomotic complications. The side-to-side technique for stapled intrathoracic anastomosis is illustrated in Fig. 6.

## Discussion

Despite the new developments (e.g., minimally invasive surgery), esophagectomy for cancer is still associated with a significant risk of perioperative morbidity and mortality [1, 4]. To reduce morbidity and mortality, it is important at the end of the procedure to create a safe gastric conduit-esophageal anastomosis with low risk of leakage.

With the increase of gastroesophageal junction tumors, the Ivor Lewis type of resection with intrathoracic anastomosis is increasingly being used. A shorter gastric conduit will permit an extended gastric resection and a well-vascularized anastomosis. Moreover, minimally invasive esophageal resection is increasingly implemented with potential benefits of having less pain, less respiratory infection, and reduced intensive care unit stay, preserving the completeness of the resection. The combination of Ivor Lewis esophagectomy with minimally invasive surgery has the potential to improve the postoperative outcome.

There is more interest for stapler esophageal anastomosis in literature than handsewn techniques [7, 26]. Stapled anastomosis in the thoracic cavity has been supported by Blackmon et al. [7] who analyzed three techniques of intrathoracic esophagogastric anastomosis: handsewn, circular stapled, and side-to-side stapled anastomosis. In this matched analysis, no significant differences were reported concerning anastomotic leakage. However, a higher incidence of dysphagia and a fourfold higher incidence of stricture were seen after the handsewn technique. Moreover, no differences were reported for both circular stapled and side-to-side stapled methods. This study suggests a clearly superior role of the stapler technique for gastroesophageal anastomoses.

This review summarizes the different techniques used to perform a safe intrathoracic anastomosis after an Ivor Lewis thoracoscopic procedure. None of the techniques described here are found superior to the others, but stapled anastomosis offered a safe outcome with a low percentage of anastomotic leakage and stenosis. Furthermore, no important differences were found between the two most used stapled anastomoses: the transoral introduction of the anvil, and the transthoracic introduction. Clinical trials are needed to compare different methods to improve the quality of the intrathoracic anastomosis after esophagectomy for cancer.
